# Astaxanthin alleviates cerebral edema by modulating NKCC1 and AQP4 expression after traumatic brain injury in mice

**DOI:** 10.1186/s12868-016-0295-2

**Published:** 2016-08-31

**Authors:** Mingkun Zhang, Zhenwen Cui, Hua Cui, Yang Cao, Chunlong Zhong, Yong Wang

**Affiliations:** 1 Department of Neurosurgery, Ren Ji Hospital, School of Medicine, Shanghai Jiao Tong University, 160 Pujian Road, Shanghai, 200127 China; 2 Department of Neurosurgery, The Affiliated Hospital of Qingdao University, Qingdao, 266005 China

**Keywords:** Astaxanthin, Traumatic brain injury, Aquaporin-4, Na^+^–K^+^–2Cl^−^ co-transporter-1, Cerebral edema

## Abstract

**Background:**

Astaxanthin is a carotenoid pigment that possesses potent antioxidative, anti-inflammatory, antitumor, and immunomodulatory activities. Previous studies have demonstrated that astaxanthin displays potential neuroprotective properties for the treatment of central nervous system diseases, such as ischemic brain injury and subarachnoid hemorrhage. This study explored whether astaxanthin is neuroprotective and ameliorates neurological deficits following traumatic brain injury (TBI).

**Results:**

Our results showed that, following CCI, treatment with astaxanthin compared to vehicle ameliorated neurologic dysfunctions after day 3 and alleviated cerebral edema and Evans blue extravasation at 24 h (p < 0.05). Astaxanthin treatment decreased AQP4 and NKCC1 mRNA levels in a dose-dependent manner at 24 h. AQP4 and NKCC1 protein expressions in the peri-contusional cortex were significantly reduced by astaxanthin at 24 h (p < 0.05). Furthermore, we also found that bumetanide (BU), an inhibitor of NKCC1, inhibited trauma-induced AQP4 upregulation (p < 0.05).

**Conclusions:**

Our data suggest that astaxanthin reduces TBI-related injury in brain tissue by ameliorating AQP4/NKCC1-mediated cerebral edema and that NKCC1 contributes to the upregulation of AQP4 after TBI.

## Background

Traumatic brain injury (TBI) is the foremost cause of neurological dysfunction and mortality worldwide and leads to substantial economic and social burdens [1]. The pathophysiology of TBI involves several complex cellular and molecular processes of primary and secondary mechanical events. Secondary events, such as cerebral ischemia, hypoxia, and brain edema, are often the main determinants of neurological outcomes and survival after TBI [2].

Brain edema is a common consequence of TBI, and two main types of edema generally occur following TBI: cytotoxic and vasogenic brain edema. Cytotoxic edema is associated with the failure of ATP-dependent Na^+^/K^+^-pumps during energy shortage and the subsequent increase in water content within the intracellular compartment in response to an osmotic gradient, which results in cell swelling. Vasogenic edema occurs when the blood–brain barrier (BBB) becomes leaky, permitting an influx of plasma constituents from the vasculature into the extracellular space [3]. Unfortunately, the mechanisms causing TBI-associated brain edema are not fully understood, thereby constraining the development of novel treatment options for brain-injured patients [4, 5]. Recent studies have elucidated the role of aquaporins (AQPs) and Na^+^–K^+^–2Cl^−^ co-transporters (NKCCs) in inducing brain edema. AQPs, a family of water-channel proteins, permit selective, bidirectional water movement in response to osmotic gradients [6, 7]. AQP4 is the main isoform expressed on ependymal cells and astrocytes and is implicated in cerebral edema induced by TBI [8]. Two Na^+^–K^+^–2Cl^−^ co-transporter isoforms, NKCC1 and NKCC2, play important roles in cellular ionic homeostasis and the subsequent accumulation of intracellular water [9]. NKCC1 is expressed in neurons and astrocytes throughout the brain and is also implicated in cerebral edema induced by TBI [9–11].

Astaxanthin (ATX), a naturally occurring carotenoid, is widely distributed in algae, crustaceans, shellfish, and various plants [12]. It was approved by the US Food and Drug Administration as a feed additive in 1987 and a dietary supplement in 1999 [13]. Increasing evidence has indicated that ATX has a variety of pharmacological properties, including antioxidative, anti-inflammatory, antitumor, and immunomodulatory activities [14, 15]. In the central nervous system (CNS), ATX is regarded as a potential drug for cerebral ischemia injury and subarachnoid hemorrhage (SAH) due to its powerful antioxidant and anti-inflammatory properties [16–19]. However, no study has investigated the effects of ATX on experimental TBI. Thus, the aim of the present study was to determine whether ATX is neuroprotective and improves the neurological deficits after TBI.

## Methods

### Surgical procedures and controlled cortical impact (CCI) injury model

The animal studies were approved by the Institutional Animal Care and Use Committee of Shanghai Jiao Tong University School of Medicine. As previously described, a controlled cortical impact (CCI) model was used in this study [20, 21]. Briefly, male C57BL/6 mice, 10–12 weeks old and weighing 18–22 g, were anesthetized via intraperitoneal injection of 100 mg/kg ketamine and 10 mg/kg xylazine. After placement in a stereotactic frame (Stoelting, Wood Dale, IL, USA), the head was shaved and then sterilized with povidone–iodine. A midline scalp incision (8 mm) was made, the skin and periosteum were retracted from the skull surface, and a 4-mm craniotomy was performed in the right parietal bone midway between the bregma and the lambda with the medial edge 2 mm lateral to the midline, without disrupting the dura. A PinPoint™ (Model PCI3000) controlled cortical impact device (Hatteras Instruments Inc., Cary, NC, USA) attached to a 3.0-mm rounded metal tip that was angled such that it was positioned vertical direction to the surface of brain was used to deliver a single impact (impact speed: 3.0 m/s, injury depth: 1.0 mm, dwell time: 180 ms), mimicking a moderate TBI in humans. After injury, the scalp was sutured closed, and the mouse was removed from the stereotaxic holder to recover. Sham-operated mice underwent an identical procedure, including the anesthesia and craniotomy, but the impact was not induced. Throughout all procedures, body temperature was monitored using a rectal thermometer and was maintained at 37 °C on a heating pad.

### Drugs and experimental groups

Mice were randomly assigned to sham, vehicle-treated, and astaxanthin-treated groups. Astaxanthin (Santa Cruz Biotechnology, USA, 98 % pure) was dissolved in corn oil (1 mL/kg) immediately before use. Thirty minutes after induction of CCI, 4 doses (10, 25, 50, or 100 mg/kg body weight) of astaxanthin were administered via intraperitoneal injection. The doses of astaxanthin used here followed the doses used by Zheng et al. [22] with some modification. Control mice were treated with an intraperitoneal injection of an equal volume of corn oil 30 min after CCI.

To further elucidate the relationship between NKCC1 and AQP4 in TBI-induced brain edema, the NKCC1-specific inhibitor bumetanide (BU) (Sigma-Aldrich, St. Louis, MO, USA) was administered intravenously (10 mg/kg body weight, as previous described [23]) 20 min before CCI. As a control, mice received intravenous injections of equal volumes of saline solution.

### Behavioral testing

The neurological functions were evaluated on days 1, 3, 7, and 14 post-CCI based on the Garcia Score. This scoring system consists of six tests, including spontaneous activity, spontaneous movement of the four limbs, forepaw outstretching, reaction to touch, climbing, and vibrissae touch. Each test is scored on a range of 0–3, and the maximal score is 18. The mean of the neurologic scores evaluated by two blinded observers was used.

Motor coordination and balance were evaluated using the rotarod test. Before CCI induction, mice were initially trained to maintain themselves on the rotating rod at 10 rotations per minute (RPM) for 120 s (habituation phase). After mice were habituated to running on the rod, the speed was accelerated to 40 RPM for 300 s. The average time to falling of the final three trials was recorded as the baseline latency. After CCI, the mice were tested 3 times per day on days 1, 3, 7, and 14, and the average latency to falling was recorded.

The Morris water maze (MWM) was employed to assess the learning and memory function of mice by training mice to locate a hidden, submerged platform based on visual information and was conducted exactly as described previously [20, 24]. The test was conducted 10–14 days after injury, and each mouse was tested for four trials per day for three consecutive days prior to injury or sham-injury. Behavioral performances for each day consisted of the mean escape latency and swim path distance. Data from all trials were recorded using a computerized video motion analysis system (Mobiledatum, Shanghai, China).

### Assessment of brain water content

Brain water content, a sensitive measure of cerebral edema, was estimated using the wet-dry method. Briefly, mice were decapitated under deep pentobarbital anesthesia at 24 h after CCI. The brains were harvested, the most rostral and caudal areas and cerebellum were discarded, and the ipsilateral and corresponding contralateral cortex were separated. Tissue samples were immediately weighed to determine wet weight, then dehydrated in a vacuum oven at 100 °C for 48 h to obtain the dry weight. Percent brain water content was calculated according to the following formula: % brain water content = [(wet weight − dry weight)/wet weight] × 100 %.

### Assessment of Evans blue extravasation

Blood–brain barrier (BBB) disruption was evaluated using Evans blue dye (Sigma Aldrich, St Louis, MO, USA) extravasation. Briefly, at 24 h post-CCI, Evans blue dye (2 %, 4 mL/kg) was injected and administered over 2 min into the left internal jugular vein, after which it was allowed to circulate for 2 h. Under anesthesia, mice were perfused with phosphate-buffered saline through the left ventricle, then the brains were removed and divided into two hemispheres to evaluate the dye extravasation. Each sample was immediately weighed, homogenized in 1 mL 50 % trichloroacetic acid solution, and centrifuged at 15,000*g* for 30 min. Then, the supernatant was diluted to 1:3 with ethanol, and its absorbance was determined at 610 nm using a spectrophotometer (BioTek, Winooski, VT, USA). The amount of Evans blue dye was calculated using a standard curve and expressed as micrograms per gram of brain tissue.

### Relative quantitative real-time PCR analysis

Total RNA was extracted 24 h after CCI from the ipsilateral hemispheres using Trizol reagent (Invitrogen, USA). Reverse transcription was performed using a Prime-Script RT reagent kit (TaKaRa Bio Inc., China). The primers used to amplify the target genes were as follows: AQP4: 5′-CTGGAGCCAGCATGAATCCAG-3′ (forward), 5′-TTCTTCTCTTCTCCACGGTCA-3′ (reverse); NKCC1: 5′-TGATTCCACTTCCTTTATTGCAG-3′ (forward), 5′-TTAATGAGTTGAGCTCCGGTGA-3′ (reverse); GADPH: 5′-AGGTCGGTGTGAACGGATTTG-3′ (forward), 5′-TGTAGACCATGTAGTTGAGGTCA-3′ (reverse). All primers were constructed by Invitrogen Corp (Shanghai). After 40 cycles, the relative levels of gene expression were calculated using SDS software (Applied Biosystems, HT7900, USA).

### Western blot analysis

Proteins were extracted from the peri-contusional cortex at 24 h post-CCI, placed in complete RIPA buffer, sonicated, and then centrifuged for 5 min at 14,000*g* at 4 °C. Protein concentrations were quantified via the BCA method using a bicinchoninic acid protein assay kit (Pierce Biotechnology, USA). Then, 50 μg of protein was loaded onto a 6–10 % polyacrylamide gel for electrophoresis, and then electrotransferred onto a polyvinylidenedifluoride (PVDF) membrane. Blots were blocked with 5 % non-fat milk for 1 h at room temperature, and incubated overnight at 4 °C in the following primary antibodies: AQP4 (1:500, sc-20812, Santa Cruz Biotechnology, USA), NKCC1 (1:100, AB 3560P, Millipore, USA), β-actin (1:1000, 8457, Cell Signaling Technology, USA), and β-tubulin (1:1000, 2128, Cell Signaling Technology, USA). After rinses with Tris-buffered saline with 0.1 % Tween-20, the blots were incubated with secondary antibodies for 1 h at room temperature. Immunoblots were then reacted with SuperSignal West Pico Substrate (ThermoFisher Scientific, USA). Chemiluminescence was detected using an imaging system, and a densitometry analysis was performed using Quantity One software.

### Immunohistological staining

After fixing with 4 % paraformaldehyde (PFA) in 0.1 M phosphate buffer, brain samples were dehydrated in 30 % sucrose solution in phosphate-buffered saline (PBS) overnight. Whole brains were then frozen in a cryopreservative solution on dry ice and coronal sections at 20 μm thickness that included the peri-contusional cortex were collected for immunohistological staining. Sections were then washed, blocked in 10 % bovine serum albumin (BSA) for 30 min, and incubated overnight at 4 °C with the following primary antibodies: rabbit anti-AQP4 (1:200, sc-20812, Santa Cruz Biotechnology, USA), rabbit anti-NKCC1 (1:50, AB 3560P, Millipore, USA), and mouse anti-glial fibrillary acidic protein (GFAP) (1:200, MAB360, Millipore, USA). After washing, the sections were then incubated with the following secondary antibodies for 1 h: AlexaFluor 488 donkey anti-rabbit IgG (1:500, A21206, Invitrogen, USA) and Alexa Fluor 594 donkey anti-mouse IgG (1:500, A21203, Invitrogen, USA). Microvessels were identified by rat anti-CD31 (1:100, 553370, BD Biosciences, USA) incubated with the primary antibody. Fluorescence images were acquired using a confocal laser-scanning microscope or fluorescence microscope.

### Statistical analysis

All data are presented as the mean ± SE and were analyzed using GraphPad Prism (version 5.01, GraphPad Software, San Diego, CA, USA). Differences among groups were assessed by a one-way analysis of variance followed by Student–Newman–Keuls test or a two-way analysis of variance followed by Bonferroni post hoc tests. A *p* value of <0.05 was considered statistically significant.

## Results

### Astaxanthin ameliorates neurological deficits following CCI

**Fig. 1:**
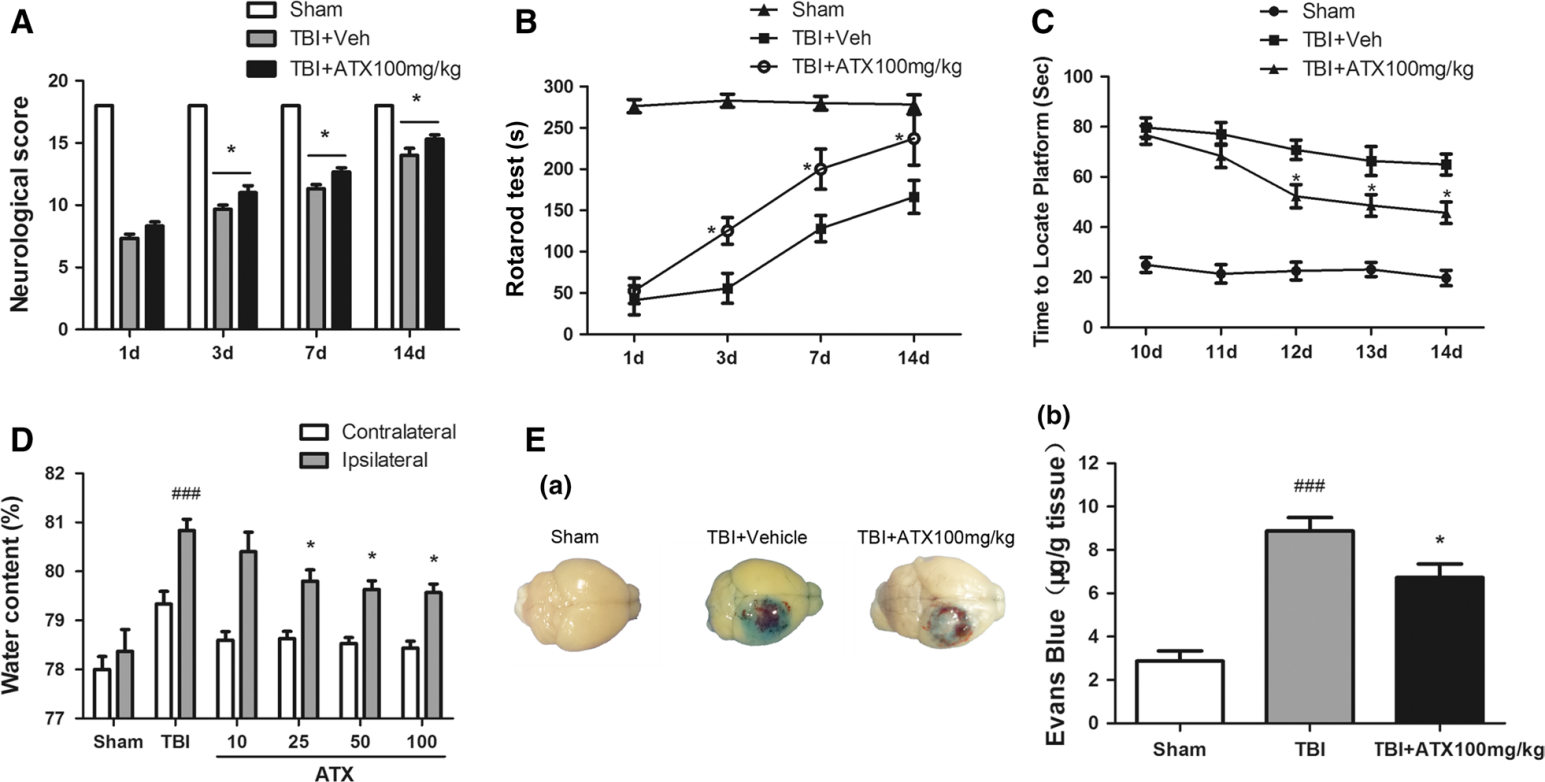
Astaxanthin ameliorated neurologic dysfunctions and alleviated cerebral edema and Evans blue extravasation. **A**–**C** Significant improvements in the Garcia score and the rotarod assessment were observed in the astaxanthin-treated group (100 mg/kg) on days 3, 7, and 14 compared to the vehicle-treated group. Similarly, the astaxanthin-treated group exhibited improved spatial learning evaluated by the Morris water maze on days 12, 13, and 14. n = 6 for each group. **D** Astaxanthin alleviated cerebral edema in a dose-dependent manner at 24 h post-CCI. n = 6 for each group. **E** Images of dye extravasation in the vehicle-treated group; administration of astaxanthin (100 mg/kg) remarkably reduced Evans blue content in the ipsilateral cortex at 24 h following CCI compared to the vehicle-treated group. n = 6 for each group. Values are the means ± SEMs. *p < 0.05, astaxanthin-treated group versus vehicle-treated group. ^###^ *p* < 0.001, vehicle-treated group versus sham group

Neurological deficits were present in all mice after CCI (Fig. 1A–C). As shown in Fig. 1A, the score in sham group was 18 based on the Garcia scoring system. A significant reduction in Garcia score after CCI was observed in all mice. However, administration of 100 mg/kg astaxanthin 30 min post-CCI markedly improved behavioral outcomes on days 3 (11.0 ± 0.6 vs. 9.7 ± 0.3, *p* < 0.05, n = 6), 7 (12.7 ± 0.3 vs. 11.3 ± 0.3, *p* < 0.05, n = 6), and 14 (15.3 ± 0.3 vs. 14.0 ± 0.6, *p* < 0.05, n = 6) compared to the vehicle-treated group.

Similarly, for the rotarod test, significant improvements were detected in the astaxanthin-treated group (100 mg/kg) on days 3 (125.32 ± 16.09 vs. 55.67 ± 18.09, *p* < 0.05; sham group: 283.10 ± 7.94 s; n = 6), 7 (200.01 ± 24.31 vs. 128.03 ± 15.95 s, *p* < 0.05; sham group: 280.02 ± 8.39 s; n = 6), and 14 (237.01 ± 32.02 vs. 166.31 ± 19.92 s, *p* < 0.05; sham group: 278.33 ± 11.78 s; n = 6) compared to the vehicle-treated group.

Moreover, after administration with 100 mg/kg astaxanthin, the TBI mice had significantly shorter latencies to find the hidden platform on days 12 (52.33 ± 4.59 vs. 70.81 ± 3.87 s, *p* < 0.05; sham group: 22.50 ± 3.52 s; n = 6), 13 (48.60 ± 4.33 vs. 66.37 ± 5.79 s, *p* < 0.05; sham group: 23.13 ± 2.83 s; n = 6), and 14 (45.73 ± 4.28 vs. 64.97 ± 4.16 s, *p* < 0.05; sham group: 17.90 ± 3.06 s; n = 6) compared to the vehicle-treated group. There were no differences in swim speed between sham and any of the injured, treated groups (sham group: 18.15 ± 3.38 cm/s; vehicle-treated group: 19.34 ± 4.13 cm/s; astaxanthin-treated group: 17.07 ± 3.55 s, *p* > 0.05, n = 6).

### Astaxanthin attenuated cerebral edema after CCI and protected BBB disruption

Cerebral edema was significantly increased in the ipsilateral cortex by 24 h post-CCI (80.83 ± 0.23 % after CCI vs. 78.37 ± 0.45 % in sham, *p* < 0.001, n = 6). After treatment with astaxanthin, brain water content was reduced in a dose-dependent manner, and doses of 25, 50, and 100 mg/kg were able to significantly alleviate cerebral edema (79.80 ± 0.23, 79.63 ± 0.17, and 79.57 ± 0.18 %, respectively; *p* < 0.05, n = 6) compared to vehicle. There was no significant difference between any of the treatment groups within the contralateral cortices (78.60 ± 0.17, 78.63 ± 0.14, 78.53 ± 0.12, 78.43 ± 0.15 vs. 79.33 ± 0.26 % in CCI, *p* > 0.05; sham group: 78.01 ± 0.27 %; n = 6) (Fig. 1D).

Changes in the BBB permeability of the ipsilateral hemisphere at 24 h post-CCI in the mice are shown in Fig. 1E. Evans blue extravasation in the CCI group was markedly aggravated compared to the sham group (8.88 ± 0.62 vs. 2.88 ± 0.47, *p* < 0.001, n = 6), and it was significantly lower in the astaxanthin-treatment group (100 mg/kg) than the CCI group (6.72 ± 0.63 vs. 8.88 ± 0.62, *p* < 0.05, n = 6).

### Astaxanthin modulated AQP4 and NKCC1 expression

**Fig. 2:**
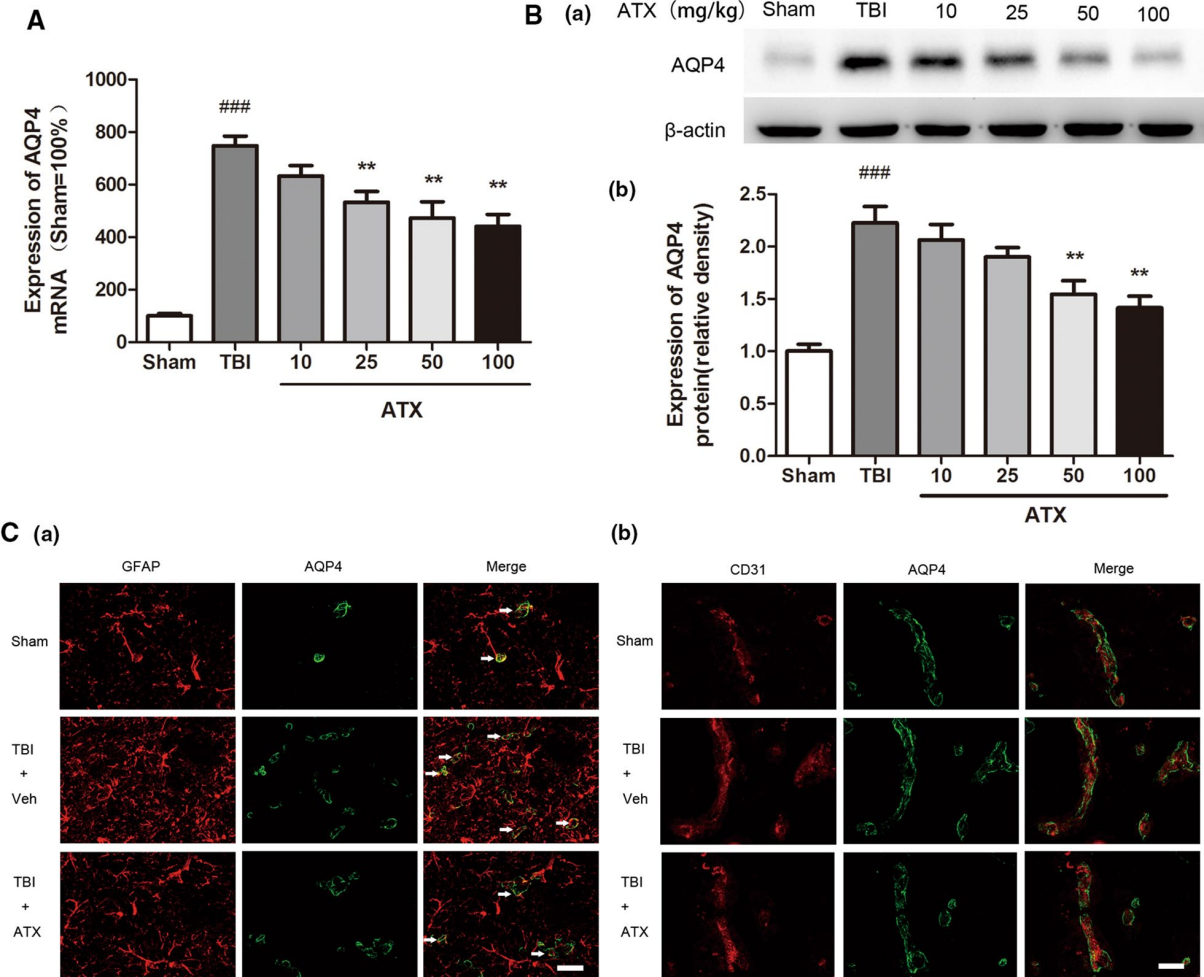
Astaxanthin reduced the gene transcription and protein expressions of AQP4. **A** AQP4 mRNA levels were significantly increased at 24 h post-CCI. Astaxanthin treatment markedly reduced its levels. n = 6 for each group. **B** The results of the western blot analysis showed that AQP4 protein expression was strongly induced at 24 h after CCI. Astaxanthin treatment inhibited its expression in a dose-dependent manner compared to vehicle treatment. n = 3 for each group. **C** Immunofluorescence staining showed that AQP4 expression was significantly increased in the peri-contusional area at 24 h after CCI, especially in the perivascular astrocyte endfeet (*white arrows*). Astaxanthin treatment (100 mg/kg) reduced its expression compared with vehicle treatment. *Scale bar* 10 μm. n = 3 for each group. Values are the means ± SEMs. **p < 0.01, astaxanthin-treated group versus vehicle-treated group. ^###^ *p* < 0.001, vehicle-treated group versus sham group

**Fig. 3:**
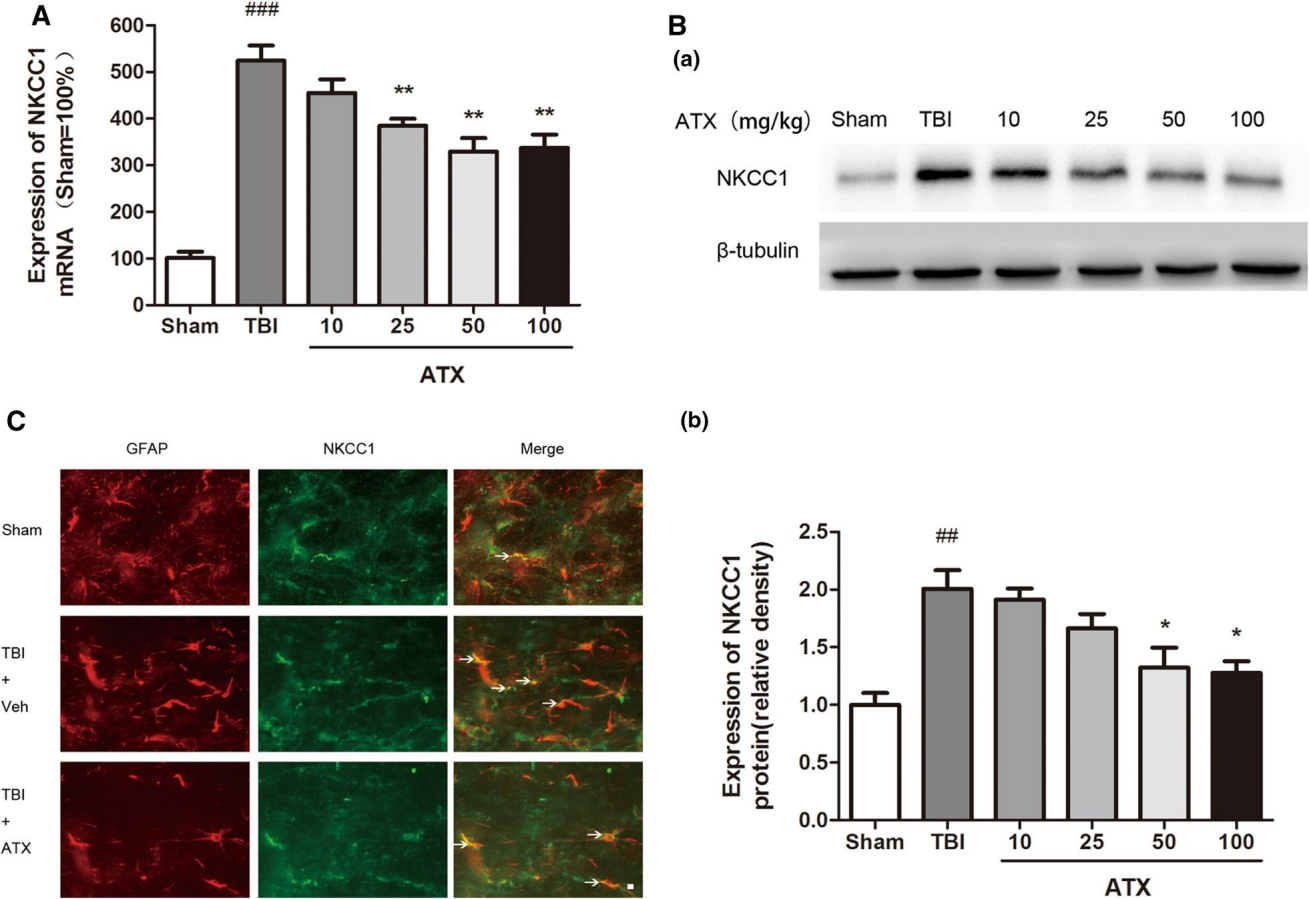
Astaxanthin reduced the gene transcription and protein expressions of NKCC1. **A** NKCC1 mRNA levels were significantly increased at 24 h post-CCI. Astaxanthin treatment remarkably reduced its levels. n = 6 for each group. **p < 0.01, astaxanthin-treated group versus vehicle-treated group. ^###^ *p* < 0.001, vehicle-treated group versus sham group. **B** Immunoblot analysis of NKCC1 showed that NKCC1 protein expression was strongly induced at 24 h after CCI. Astaxanthin treatment inhibited its expression in a dose-dependent manner compared to vehicle treatment. n = 3 for each group. *p < 0.05, astaxanthin-treated group versus vehicle-treated group. ^##^ *p* < 0.01, vehicle-treated group versus sham group. **C** Representative immunofluorescence staining of NKCC1 showed that NKCC1 markedly increased in the peri-contusional area at 24 h after CCI (*white arrows*). Astaxanthin treatment (100 mg/kg) reduced its expression compared with vehicle treatment. Scale bar = 10 μm. n = 3 for each group. Values are the means ± SEMs

As shown in Figs. 2A and 3A, AQP4 and NKCC1 mRNA were elevated within the peri-contusional cortex at 24 h (748.30 ± 36.16 and 524.01 ± 32.91, respectively; *p* < 0.001, n = 6) following CCI. They were attenuated by astaxanthin in a dose-dependent manner at 24 h, and high doses of astaxanthin (25, 50 and 100 mg/kg) reduced their mRNA expression remarkably compared to vehicle (532.71 ± 41.26, 473.02 ± 61.88, 441.04 ± 45.24 for AQP4, respectively, *p* < 0.01; 384.72 ± 14.88, 329.04 ± 29.44, 337.03 ± 28.59 for NKCC1, respectively, *p* < 0.01, n = 6).

Similar to the results obtained by mRNA, the protein expressions of AQP4 and NKCC1 were strongly increased at 24 h after CCI (2.23 ± 0.16 vs. 1.01 ± 0.06 and 2.01 ± 0.16 vs. 1.02 ± 0.10, respectively, *p* < 0.001, n = 3), and these inductions were significantly reduced following a single injection of astaxanthin at doses of both 50 mg/kg and 100 mg/kg (1.55 ± 0.13 and 1.42 ± 0.11, respectively, for AQP4, *p* < 0.01; 1.33 ± 0.17 and 1.28 ± 0.09, respectively, for NKCC1, *p* < 0.05, n = 3) (Figs. 2B, 3B).

Immunofluorescence staining for AQP4 at 24 h after CCI showed that AQP4 expression was significantly increased in the peri-contusional area, especially in perivascular astrocyte endfeet (Fig. 2Ca, b, n = 3). Administration of astaxanthin (100 mg/kg) decreased AQP4 expression compared to the vehicle-treated group (n = 3). A similar effect was observed for NKCC1 expression: immunofluorescence showed that its expression was markedly increased in astrocytes in the peri-contusional area and that this effect was inhibited by astaxanthin administration (100 mg/kg) (Fig. 3C, n = 3).

### Inhibition of the NKCC1 downregulates trauma-induced AQP4 expression

**Fig. 4:**
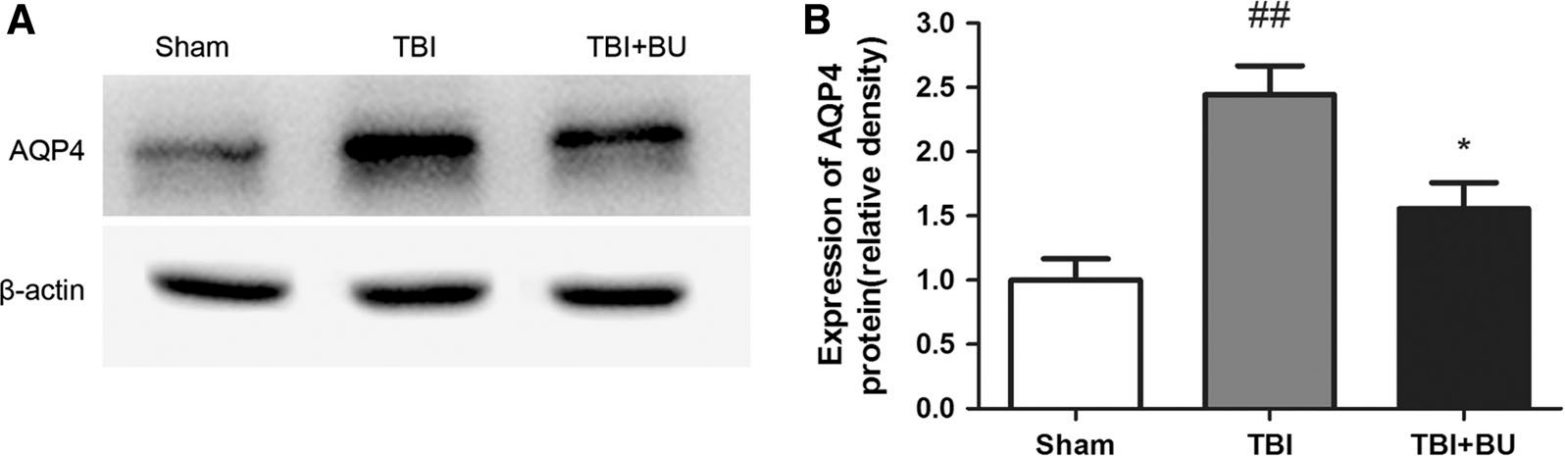
Bumetanide (BU) inhibited trauma-induced AQP4 expression. **A** The protein expression of AQP4 was illustrated by western blot analysis. **B** The immunoreactivity of AQP4 was significantly increased at 24 h after CCI. A single injection of bumetanide (10 mg/kg) reduced its upregulation. n = 3 for each group. Values are the means ± SEMs. *p < 0.05, bumetanide-treated group versus vehicle-treated group. ^##^ *p* < 0.01, vehicle-treated group versus sham group

Western blot analysis illustrated that AQP4 expression was markedly elevated at 24 h after CCI (2.45 ± 0.22 vs. 1.00 ± 0.16, *p* < 0.01, n = 3) compared to the sham group. Treatment with bumetanide (BU, 10 mg/kg), an inhibitor of NKCC1, significantly inhibited the trauma-induced AQP4 upregulation (1.56 ± 0.20 vs. 2.45 ± 0.22, *p* < 0.05, n = 3) (Fig. 4).

## Discussion

The present study showed that astaxanthin improved neurological outcome following TBI by alleviating cerebral edema in a dose-dependent manner. This effect was correlated with decreased peri-contusional expressions of AQP4 and NKCC1. In addition, we demonstrated that NKCC1 inactivation influenced the expression of AQP4. To the best of our knowledge, our results illuminate, for the first time, the relationship between astaxanthin treatment and the extent of cerebral edema in a TBI model. Thus, astaxanthin may represent a novel therapeutic agent for anti-edema drugs after TBI.

Cerebral edema and the ensuing increased intracranial pressure are major factors leading to the high mortality and long-term disability of patients [25, 26]. Intracranial edema can be classified into two main categories: cytotoxic edema (intracellular edema) and vasogenic edema (extracellular edema) [27]. Cytotoxic edema commences within the first hours after injury and dominates in the early phase (2–24 h) [28], during which astrocytes are the principal cells affected. Disruption of the BBB produces vasogenic edema, which increases 24 and 72 h after TBI and disappears 2 weeks after the lesion [29]. In our study, brain edema was evaluated at 24 h, a timepoint that has been reported as the maximum following TBI in mice [30]. We found that astaxanthin reduced cerebral edema significantly at 24 h. Combined with the results of the Evans blue measurement, we speculated that astaxanthin may exert a protective effect on cerebral edema by decreasing both cytotoxic edema and vasogenic edema. Furthermore, we demonstrated that cerebral edema was attenuated by astaxanthin administration in a dose-dependent manner. Coupled with a report suggesting that rat oral LD_50_ values of astaxanthin are 12 g/kg body weight [31], the relatively high dose (100 mg/kg) administered to the mice is safe for reducing cerebral edema and improving neurological performance following TBI.

As a therapeutic agent with potential clinical applications, the feasibility of a wider therapeutic window for prophylactic treatment is inapplicable because of the unpredictability of TBI. Astaxanthin is considered to cross BBB easily and rapidly [32] due to its lipophilic nature [33]. In our study, astaxanthin administered 30 min after TBI significantly reduced cerebral edema at 24 h. Zhang et al. [18] reported that oral administration of astaxanthin at 3 h post-SAH significantly reduced brain edema and Evans blue extravasation at 24 h in rats. Future research is needed to identify whether it is effective when administered at different time points. Shen et al. [16] demonstrated that astaxanthin injected into the lateral ventricle can be effectively distributed onto the cortical surface and reduce ischemic brain injury in rats, implying that improvement of administration route will increase the protective efficiency of astaxanthin. Integrating astaxanthin into additional forms, such as nanoparticles or liposomes, may overcome some of its limitations, such as solubility, stability, and bioavailability, and provide an extended therapeutic window for TBI treatment.

Inappropriate activation and overexpression of AQP4 and NKCC1 contribute to cell swelling and tissue edema [11, 34]. The present study suggests that the mRNA and protein expressions of AQP4 and NKCC1 are up-regulated in peri-contusional brain tissue. Double immunofluorescence stainings further demonstrated that AQP4 and NKCC1 expressions were localized in cerebral cortex astrocytes, as verified by their co-localization with GFAP, a specific cellular marker for astrocytes. More importantly, we verified that astaxanthin treatment attenuated their expressions in cerebral cortex astrocytes according to the reduction of brain water content in a dose-dependent manner. Taken together, these results suggest that astaxanthin attenuates cerebral edema by regulating AQP4 and NKCC1, although it is possible that astaxanthin reduces brain injury, which then impacts AQP4 and NKCC1 expression.

The underlying protective mechanisms of astaxanthin in the central nervous system remain unclear. Whether astaxanthin influences AQP4 and NKCC1 expressions directly or indirectly in TBI is to be further determined. It has been reported that the reduction in ischemic brain injury in adult rats due to astaxanthin involves inhibition of glutamate overflow [16]. A recent study documented that excessive glutamate levels up-regulated astrocyte AQP4 activity, which further exacerbated the edematous insult [35]. Similarly, Lu et al. [9] reported that the interaction between NKCC1 and glutamate exacerbated NKCC1 overexpression and the development of brain edema during TBI. These data suggest that astaxanthin may exert its neuroprotection on TBI by regulating AQP4 and NKCC1 expressions via the glutamate system.

In our study, we demonstrate that AQP4 upregulation is significantly inhibited by bumetanide following TBI, as previously reported [34], indicating that NKCC1 activation influences AQP4 upregulation by TBI. Although the mechanism by which NKCC1 activation causes the upregulation of AQP4 is unknown, it is presumed that a disturbance in osmotic gradients induced by increased activation of ion transporters may trigger a signaling event, culminating in the upregulation of AQP4 [36]. Nevertheless, future studies are needed to precisely delineate the mechanism by which NKCC1 contributes to the upregulation of AQP4.

## Conclusion

Our study suggests that astaxanthin may exert neuroprotection following TBI by ameliorating AQP4/NKCC1-mediated cerebral edema, and NKCC1 inactivation inhibitions the upregulation of AQP4 after TBI. Further studies are required to investigate the potential clinical efficacy of astaxanthin for TBI treatment.

## Abbreviations

AQP: aquaporin
ATX: astaxanthin
BBB: blood–brain barrier
BSA: bovine serum albumin
BU: bumetanide
CCI: controlled cortical impact
CNS: central nervous system
GFAP: glial fibrillary acidic protein
MWM: Morris water maze
NKCC: Na^+^–K^+^–2Cl^−^ co-transporter
PBS: phosphate-buffered saline
PFA: paraformaldehyde
PVDF: polyvinylidenedifluoride
RPM: rotations per minute
SAH: subarachnoid hemorrhage
TBI: traumatic brain injury
